# Intellectual Disability, Employment and Aging: Intervention Measures

**DOI:** 10.3390/ijerph18062984

**Published:** 2021-03-14

**Authors:** María Teresa Ortega-Camarero, José Luis Cuesta-Gómez, Raquel de la Fuente-Anuncibay

**Affiliations:** 1 Department of Private Law, Area of Sociology, University of Burgos, 09001 Burgos, Spain; mocamarero@ubu.es; 2 Department of Education Sciences, University of Burgos, 09001 Burgos, Spain; raquelfa@ubu.es

**Keywords:** intellectual disability, employment, aging, measures, quality of life

## Abstract

Workers living with intellectual disability suffer in a special way from the onset of premature aging. Hence the need to generate alternatives and policies for the development of a new model for active aging and the care of workers with intellectual disability. Our objective in this study is, therefore, to devise intervention measures that can minimize the effects of aging on the lives and the activities of these workers. Employing the Delphi technique, we assembled and consulted a panel of 8 experts with relevant expertise in the fields of intellectual disability; aging; employment and dependency. The panel included employers, families and workers with disability. Each expert reached a prior consensus over every response and contribution after having consulted four other experts with similar backgrounds, a consultative process in which a total of 40 experts participated. A total of 68 measures were proposed which correspond to three lines of action or key contexts: Firms and Organizations that employ People with Intellectual Disability; People with Intellectual Disability, and Family setting. In all, 10 recommendations with a focus on both firms and organizations were advanced to implement the proposed measures. The conclusion was that interventions are needed from the time at which the first symptoms of deterioration are detected, which should not necessarily lead to immediate loss of employment, as well as individualized and coordinated interventions among all relevant stakeholders, including the families.

## 1. Introduction

The aging process, although not exclusive to people with intellectual disability (PID), presents distinctive features within this specific group. Longer life spans over the last century, closely related with improvements in their quality of life, have recently awakened interest in the topic, increasing the number of studies on this topic over the past two decades.

The data from the United Nations (UN) ‘World Population Perspectives’ annual reviews between 2017 and 2019, all signal an increase in the number of people over 60 years old, which is likely to pass from 962 million to 2100 million in 2050 and even to 3100 million in 2100. Increased life expectancy in a majority of countries is also foreseen [1].

Although there is a tendency to treat older people as a homogeneous group, this group covers a broad social spectrum of very different ages and with very varied demands and needs. Aging is a biological fact, which does not mean that it must entail significant physical or psychological deterioration. Some studies have pointed out that the health of the working population will not depend so much on the most recent employment conditions, but on the conditions throughout the whole period of active employment, which makes it necessary to approach the problems of aging workers before they are around 50 to 55 years old [2].

Along these lines, the data from the Programme for the International Assessment of Adult Competencies (PIAAC), developed by the Organization for Economic Cooperation and Development (OECD) in 2013, were used to analyze the cognitive skills of individuals between 16 and 65 years of age in 34 countries. Its conclusion was that certain skills related with the capacity to carry out physical work, comprehension of reading and arithmetic and the use of new technologies all deteriorate. On the contrary, with greater experience, the older workers will develop greater capabilities for planning, supervision and reacting to unforeseen event. Although it is conditioned by many factors such as the absence of illness, maintaining an active life and the practice of physical and cognitive work can vastly improve future perspectives [3].

Brindusa and Lacuesta [3] pointed to the relation between cognitive deterioration and the level of training of the groups they studied in 16 countries outside the Euro zone. In this sense, the loss of capabilities can be delayed, because of such aspects as activities and continuous training throughout life. Some authors [4] noted that older people might be less likely to work in jobs where physical effort and higher levels of knowledge are required.

In the whole process of retirement from working life, a key aspect to take into account is the perspective of retirement and pre-retirement for people and organizations. The types of aging—normal, referring to natural deterioration over time; pathological change provoked by illness; and the optimal state—are conditioned by employment perspectives, as well as the retirement age.

In this sense, retirement is understood as a process that starts before the official age for retirement and is prolonged for some time afterwards. Facing up to this stage implies the need to promote measures for a gradual transition. The changes associated with income levels and occupational status have been pointed out. These entail significant repercussions for physical and psychological states of health [5] and one study [6] found a clear decline in wellbeing and vital satisfaction during the first six months after retirement. The importance of taking into account the different rhythms of adaptation may be highlighted, as Robert Atchley [7] mentioned, in the necessary completion of a series of phases that delink the person from the place of employment until the worker is prepared to accept the role of a retired person or pensioner.

The study completed in Italy by Chiesa and Sarcheli [8] involving 250 public employees from Italian administrations, all over 48 years old, pointed to the existence of aspects that come before retirement such as social support received both from within and outside the firm and family support that influences anxiety towards retirement, a feeling of loss of social identity, and social exclusion, which can vary the desired age of retirement.

The challenges and solutions that aging raises are of growing concern, especially within sectors linked to the field of disability [9]. It is a matter that impacts on social policies, service delivery, professional practice [10] and medical advances, partly because of the social relevance that this collective is assuming, but also because of its quantitative transcendence. Medical advances, preventive treatment, and improvements in the conditions of life and the services that they receive [11] have meant that increasing numbers of PID reach advanced ages suffering from complex deterioration within their organisms and in their lives with respect to their younger years. This calls for a reworking of the models and the intervention systems where working life is one of the priorities.

With respect to the population living with intellectual disability, according to estimates found in the study by Berjano and García [12], a total of 28,819 PID are over 45 years old, which represents 18% of the total of all PID in Spain. In the same study, a forecast of the number of PID over 45 years old in 2026 yielded an estimate of 109,881 people, which implies that 63% of the PID could have started their aging process in 2026, at all times in the absence of significant events and maintaining present-day rates of prevalence of intellectual disability. These data are corroborated by the statistics in Spain that point to 60% of PID who will be over 45 years of age within a decade [13].

The non-existence of a specific central registry on age and disability leaves us without reliable data on the number of PID in the aging process [12]. This situation is even more complex, if we approach the data in an international context, taking into account that there is no common definition of disability, not even at the level of the European Union, and that there is a great diversity of realities behind the word “disability”, which has increased with migration from Eastern European countries (2004 and 2009) [14]. In all, 45.5% of the population living with disability in Europe are undergoing an aging process, and an increase of 400% in the life expectancy of some collectives has been documented in the United States, such as people with Down’s Syndrome [11], which implies an immense demographic challenge [15].

The data referring to Spain, according to the Survey on Disability, Personal Autonomy and Situations of Dependency [16], indicated that there were 3,847,900 million residents in Spain in 2008 who affirmed that they were living with some sort of disability, which represents 8.5% of the general population. By age ranges, 37.4% were between 65 and 79 years old, representing 26.7% of the total of all people with some sort of disability. It is likely that the current data on people in employment might be higher than those figures.

At present, it is noted that the life expectancy index of PID is close to the population with no disabilities. It has generated expectations and doubts, marked by the lack of evidence on questions related with the number of people that are affected and, as Berjano and García pointed out [12], the perceptions of PID and their families with regard to the aging process and how they should face up to it; what the role of professionals should be; what the principal needs are; and what the best approach is to resource provision.

Together with increased life expectancy, our attention is drawn to the premature aging that PID experience with respect to the rest of the population and compared with other sorts of disabilities [17], and to the increased intensity of their support needs. In a systematic review, Schepens et al. [18] found 73 publications, since 2015, that presented experimental designs and analyses of the relation between specific strategies and the quality of life in old age. Their study highlighted the need to plan services and professional support through centralized planning of individual necessities, paying special attention to emotional wellbeing.

The first plans for international action within the European context go back to the 1st World Assembly on Aging held in Vienna in 1982, which acknowledged the idea that people at advanced ages should lead a full, healthy and satisfactory life [19]. However, it was not until the 2nd International Plan on Aging of the World Health Organization (WHO) that attention was, for the first time, directed towards the aging of PID as an emerging pattern that governments must take into account. Its aim was to promote the development of programs and resources for this population group. The Declaration of Graz, according to Weber and Wolfmayr [20], was the first official document that called for this sort of analysis.

The United Nations Convention on the Rights of People with Disabilities (2006) [21], in coherence with its inclusive inspiration and linked to the Model of Rights, covers the concern for this topic and defends the right of the PID to healthy aging, which according to [19] means the right to age well, with resources that guarantee personal wellbeing and maintenance of the quality of life that has been achieved.

This situation is accentuated among PID. Premature aging has a great impact on workers. They begin to undergo important changes at around 45 years old that make it difficult for them to continue performing the tasks they had been doing and that can even prevent them from working at all [12], which has immense implications for their social networks and means of subsistence.

The situation with regard to the employment of people with disability is a priority on the European social agenda [22]. There are 80 million PID in Europe and their employment rates stand at around 50% [23]. Any analysis of the situation of disability and employment in this context is complicated by the inherent conceptual boundaries of disability, which can differ from country to country, as various organizations both within Europe and in Spain have warned [24,25,26]. The lack of exact figures and statistics relating to PID [27] and an absence of common criteria within European countries complicate efficient planning of similar public policies and generate immense difficulties when comparing the reality of the different employment alternatives at a European level.

Bearing in mind the above and taking the global references relating to disability and employment in Europe as a reference, which in great measure fit the reality of the different countries, according to the most recent data available from Eurostat [24], almost 45 million people of working age (15 to 64 years old) suffer from some sort of disability. A figure that represents 14% of the population within that age cohort. Inactive people imply 46% of the total, a figure that is 20 percentage points lower than the number of inactive PID and that, without doubt, represents the principal gulf between both groups and one of the main obstacles to any progress towards greater employment opportunities. However, the probability of being employed depends on the type of disability, because those people in need of personal assistance or some type of specialized equipment to carry out their employment activity, such as PID, have less probability of finding employment, despite the similar levels of importance that are attached to other variables such as sex, age and level of training, which also condition the statistical results [24].

Faced with these data, the European Strategy for the Rights of Persons with Disability 2010–2020, [23] prioritizes employment among the 8 areas of action within its framework for action at a European level. Its objective is that as many PID as possible earn income for employment activities in the open market, contributing in that way to achieve the targets on the open labor market that the European Union has set. Among other commitments, it highlights support for the transition from education to the employment of young people with disability and to approach internal mobility in the open labor market and in sheltered workshops through information exchange and mutual learning. It is presented as an attempt to incentivize the transition, which is scarcely evident in Europe, from sheltered employment to the open labor market. In this way, the 4-year strategy of political leaders agreed upon in 2017 [22] in relation to the Rights of Disabled People, calls for secure employment with regard to the working conditions of people with disability; innovative and quality entrepreneurship (diversity charters) and the promotion of the transition to employment on the open labor market, which for Europe continues to be the desirable model, as established in the text of the UN Convention [21].

This strategy of employment in the open labor market is also prioritized in a majority of European countries where a percentage of jobs are set aside for PID, such as in both France (6%) and Italy (1%), while job placement and support services are provided to accompany PID in the employment process within various other countries, such as Germany, the United Kingdom and Sweden [28].

In 2018, people with disability (PWD) entered into a total of 339,119 contractual relations in Spain [29]. The employment rate of this population group with 43 fewer percentage points than the general population continues to be very low [30]. By type of disability, the situation for PID is immediately more complex than it is for other disabilities. According to the same source, the Spanish National Institute of Statistics, only one out of every four PID has a job. Long-term unemployment is today one of the most important obstacles to the active employment of PID [31] and priority integration can be done in two ways: contracting firms from an open market and the incorporation of specialized supports and services for PWD in firms. By age groups, the highest number of contractual relations was within the age interval 25 to 44 years old, while over 156,000 workers with intellectual disability (WID) older than 45 years of age were still employed [30].

It is important to note the employment alternatives available to PID in Spain, which differ according to the capabilities and the competencies of the workers, as well as the offer of social and employment-related networks that also have strong links with social assistance and benefits for employing these workers. There are three predominant social options: occupational centres: special employment centres and the ordinary firm that they can mainly choose through assisted employment schemes. In Spain, the predominant model for PID is the Special Employment Centre [Centro Especial de Empleo] managed by social organizations, set up to facilitate the transition towards ordinary employment, but with little movement towards the open market.

In 2019, over 70% of the specific employment contracts for PID in Spain came under this employment option [26], which highlights the difficulties surrounding the transition to the open market. There appears to be a certain consensus over the non-functionality of the Special Employment Centres, in so far as they are not functioning as a mechanism for the integration of PID into the open labor market [32,33,34], their mission having evolved from a bridge between markets to the retention of talent and the improvement of their competitiveness [35]. Some reasons that could explain this permanence in sheltered employment of the PID are a greater feeling of safety that these occupational centres provide, both for the workers themselves and for their families; [32] the lack of mechanisms for returning, if the employment experience on the open market fails and, at more advanced ages, the lack of support and social benefits for accompaniment and adaptation at retirement age.

In this sense, the entrepreneurial activity concerning sheltered employment in Spain, widely acknowledged within the sector of the Social Economy of which it forms part [36], is recasting this model, which could move towards the concept of inclusive firms, working towards the professionalization of management and technological renewal, but also towards equal pay and employee training [37], in line with the recommendations of article 27 of the UN Convention [21].

This prevalence of sheltered employment implies that aging at work for the majority of PID takes place at this sort of center. It is partly due to the real problems that increase in these people as they age. Their deterioration occurs at such an early stage and because there is neither social not legal assistance at present, it means that many people in this situation and in the face of uncertainty opt for employment situations in sheltered centres, moving away for remunerated labor on the open market. Finally, it is important, on this point, to mention Occupational Centres as a pre-employment alternative, on occasions as a place for job training, and in other cases destined towards those PID with greater levels of dependency that because of their limitations cannot access employment in any of its modalities.

Stancliffe et al. [38] observed a low presence of people with intellectual disability applying for work on the open market and among the successful job seekers, a high percentage of whom abandoned the employment market before retirement due to mobility-related problems. This situation generates the need to promote measures for the work place that prevent abandonment. Moreover, these authors outlined the need to design plans that can ensure continued employment for the workers who choose to do so, with the possibility of changes and adjustments in their workplace routines relying on personalized support.

Other studies have expressed alarm over this situation and the age-related changes for PID in employment. Most of the investigations revealed evidence of cognitive, relational, physical and mental deterioration, in people whose symptoms are present at around 45 years of age [10,12,13,14,15]. In fact, it appears that there is consensus over the onset of aging at this age. In this respect, the works of Escolar [39], Novell [14], Bayarri [40] and Elorriaga [17] all point to the effects of aging among WID in the workplace, such as increased fatigue and clumsiness; loss of higher faculties including memory, attention, and spatial-temporal orientation, as well as blunter reflexes [39]. The onset of deterioration is said to be at 30 years of age when a tendency for loss of working capabilities may be observed across the board, especially in attitude; perseverance; quality of life; polyvalence; and discipline, leading to an abrupt loss of productivity at 50 years of age [40]. The consequences of these losses for employment activity and employees are significant, because of a reduced pace of work; loss of capabilities to carry out the work and reduced motivation, with the consequent risk of occupational accidents and increased sick leave [39]. In addition, difficulties are observed over quick completion of tasks; moving to subsequent tasks when one is finished; making proper use of materials; and taking significant breaks. Likewise, the quality of their work has to be supervised, they have to be called to attention, their work is not always up to standard and they may have difficulties carrying out various tasks [40].

Age also impacts on personality traits: irritability increases and humors change; anxiety increases, as do phobias and obsessions alongside other behavioral disorders, which in a productive setting tend to intensify difficulties over relations with fellow workers that affects the atmosphere at work [39]. In short, consequences are highlighted that include the loss of productive capabilities, leading to an increase in absenteeism from work [14]. There are likewise consequences for the impact of aging on self-esteem and the possible emergence of depressive symptoms [17], to a greater or lesser degree in all the quality-of-life dimensions [41], especially emotional wellbeing, social inclusion, and interpersonal relations [42].

In view of this reality, WID face an absence of alternatives to working life and an absence of measures to follow during the aging stage. This situation is generalizable to the whole collective and is of special concern for the PID themselves and social organizations that work with disabled people [10,11,43].

Although this collective has a longer life expectancy than some years ago, very little is known about retirement-related experiences. The study completed in Norway by Engeland et al. [44], on the basis of interviews with retired PID showed that they all perceived the end of useful employment as a very complex period that is difficult to assume. The investigation concluded with the need to design individualized plans and personalized approaches, in order to facilitate this transitional stage.

The inexistence of tools and validated instruments to measure deterioration may be added to this absence of measures, both from the perspective of employment and performance, as well as the influence of the surrounding context on WID. Among various tools, the one designed by the Association Lantegui Batuak in Spain [17] implies an advance, although it presents the limitation of an exclusive focus on workplace performance and is based on the subjectivity of the observer.

In view of the situation described above and from the occupational point of view, an unresolved debate has been opened with opposing positions. On the one hand, it appears advisable to promote early retirement from work at early ages, well before the normal retirement age. However, certain experiences of that sort suggest that it is not the most suitable solution and their analysis has revealed negative consequences for people, such as a negative personal impacts that degenerate into feelings of inutility; a negative effect on social relations, especially in cases where social life is linked to the working environment; and economic problems arising from the decrease in salaried income from work, especially given the difficulties that PID have faced over the recognition of their pension rights [11,17].

Despite the interest aroused over recent years, these studies and alerts on the topic have not permeated the political reality [45,46], to the point where an approach is necessary that incorporates the proposals from the groups of the affected population and other sectors that are involved.

The following investigation of a qualitative nature was, therefore, conducted in view of the current situation described above, with a significant absence of measures, alternatives, policies and practices that respond to the consequences of premature aging and the deteriorating health of WID. Our objective in this study is to devise intervention measures that can minimize the effects of aging on the lives and the activities of these workers. Measures are proposed for WID that can guide the interventions during the last stage of their employment, both in the work environment and in the immediate context.

The study consists of two parts, in the first part we will present our design of a set indicators that has to be taken into account in the different areas, so as to stimulate the necessary changes that will serve to guide and to support their implementation in a second phase, at present under development. Although the considerations of international policies on the topic have been taken into account, the objective has been through consensus to systematize the starting point, in order to undertake subsequent improvements.

The aim is to generate objectifiable measures in the lines of action with greater impact on the lives of aging WID and firms and organizations that employ them, PID and their family environments, and in essential spaces such as housing and health, leisure and interpersonal relations.

## 2. Investigative Procedure

### 2.1. Investigation Method

The objective of the study is to define a set of measures that accompany the aging process and deterioration of WID, thereby improving their quality of life. It implies the preparation of a theoretical construct that must be validated, which is achieved here through the Delphi technique. According to Ruiz Olabuénaga [47], it is a qualitative consensus-based technique within the framework of qualitative investigation methods. It is also a sociological investigative technique that involves the in-depth interview and, more specifically, the in-depth group interview.

This technique begins with the fundamental assumption that the opinion of a single individual is less reliable than the opinions of a group of people under equal conditions. The technique is, therefore, nourished through knowledge and the opinions of people who can be qualified as experts. The Delphi technique gives insight into or measures the degree of consensus between different aspects or orders them in a hierarchy in accordance with their importance and the significance that the experts attribute to each aspect [46]. The information is obtained through consultations with experts from one field or area and the definition of the aspects in which a higher level of consensus is shown [45,46].

Various authors [48] defines this technique as an iterative process, normally of three or more consultations formulated through questions. Each one is based on the results of the previous consultation, the purpose of which is open exploration of a topic until a consensus is reached through the repeated contributions of the whole group.

The qualitative investigation method was chosen, on the understanding that it fits our objective better, because of its characteristics that Mucchinelli [49] defined: it is conceived from a comprehensive viewpoint; it approaches the object of study in an open and broad manner; the ideas and concepts that underlie our proposal and that are the objective of its design and validation cannot be easily quantified or transformed into numerical variables for their analysis; and, finally, the aim is to arrive at a theoretical development. In brief, this investigation is approached through its objective that is focused on knowing the personal opinions and beliefs, thoughts on reality, and proven evidence that emerge from both working and personal experiences [50].

From a methodological point of view, the Delphi technique is a relatively flexible strategy that has allowed us to act independently and to adapt its habitual dynamic to the objectives of our investigation. The starting point for setting up this strategy was the existence of a problem that might require the opinion of a group of experts whose knowledge of the topic, characteristics, and experience is estimated a priori as appropriate for the achievement of our objectives [51].

This technique represents a fundamental step in the methodological design [52] and it means that a proposal for intervention measures may be constructed on the basis of the evidence that has been obtained in earlier quantitative investigations referring to the impact of aging on the quality of life of workers living with intellectual disability (ID) [42].

Its advantages are as follows. It guarantees the anonymity of the intervening parties and, therefore, the liberty of their contributions. It involves controlled feedback and likewise guarantees the statistical response of the group. It facilitates the representativeness of the most significant interest groups on the matter, represented by a group of heterogeneous experts linked with the life of the PID who are aging. Triangulation may be applied, contributing various perspectives [53]. It also guarantees the independence of the participants.

### 2.2. Participants

A group of 8 experts was formed who together contributed different perspectives, both on the problem and on the proposed measures, and whose expertise was related to different PID-related areas of intervention.

With a view to countering any possible inherent bias among the facilitators, each expert agreed to compile and to reach a consensus over the information to be facilitated in their field in the different phases of the technique, with at least 4 sub-experts. Data protection and confidentiality was guaranteed at all times.

In all, 62% of participants were male and 32% were female, aged between 45 and 68 years old. In relation with the years of experience in the field of disability, 70% of participants had over 15 years professional experience, 20% of participants had past experience ranging between 15 and 7 years, and 10% had less than 7 years. With regard to the professional profiles, 25% of participants were employers and managers of firms that contract PID; 25% were technicians linked to ID, employment and aging; 25% were researchers from the social sector and universities; 10% belonged to the area of families, and 15% of participants were WID in employment; 75% of participants held higher education qualifications in economics, teaching, pedagogy, psychology and law, as well as having followed training courses on disability, employment, aging and the management of organizations in the service sector.

The criteria for their selection were as follows: their special link with the topics of disability; aging; employment; and social and health care, adding to the earlier criteria those that in our opinion the experts were expected to meet to form part of the panel, which were:

1. Broad knowledge of the sector of intellectual disability and development.
2. Comparative experience or working relation of over five years within this field.
3. A multi-disciplinary nature that contributes the perspectives of different interest groups.
4. Territorial diversity.
5. Objectivity.
6. The participation of a person with intellectual disability was also ensured.

The selection of the eight experts was, therefore, an essential process, in order to guarantee the effectiveness of the technique and, in our case, the success of the resulting intervention measures. In all cases, there were certain conditions with which the experts had to comply and that were guaranteed throughout the selection process of our panel: willingness to participate; commitment towards the activity; availability over time; and communicative capability [54].

Work was conducted with all the questionnaires and materials that formed part of the different consultation phases to adapt and to simplify them for easy reading, so that the person with ID sitting on the panel of experts could clearly understand them. A professional copy editor with no previous links to the team of investigators completed the editing process for easy reading and assisted with the process of analyzing the documents and the statements of opinion.

### 2.3. Design and Validation Process of the Intervention Measures

The first consultations were circulated, once the group of experts had been assembled, having personally contacted the experts and informed them of the objectives of the investigation, which motivated their participation. The consultations were in writing, through e-mails to each expert, who responded in an individualized way to the researchers, thereby ensuring confidentiality and freedom of opinion. The four consultations that took place were sufficient to construct the proposed measures, as well as the recommendations for their subsequent application. In each consultation, the results were discussed within the panel, for the validation of each step. Each expert received individual feedback on how their contributions had been considered.

The first consultative phase that began the investigation pursued the two following objectives:

1. To reach a consensus over the areas that should be included as the key areas, in which the specific measures that target WID will subsequently be incorporated.
2. To reach a consensus over the definition of each area.

Eight areas for intervention were proposed for their validation by the experts that had been designed on the basis of the results of a previous investigation concerning the impact of age on the quality of life of the 49 workers, through the application of the INICO-FEAPS scale, documental analysis and the previous experience of the researchers in this sector. The opportunity was offered to each expert to propose other areas that might be considered significant and that had not been included in the consultation.

The experts had to value each of the areas, scoring them on a scale of 1 to 5, in which 1 was considered not very important and 5 very important. The possibility was available to include observations within each area. Table 1. represents the areas and their explanation, as assessed by the experts.

In a second consultation, having received all the contributions from the experts, each one was categorized and ordered, incorporating the new contributions from the group that reached a higher level of consensus. The experts were informed of how all their contributions from the first consultation had been taken into account.

This second phase also had as its objective the validation of the new structuring of the areas in the proposal for intervention measures that, following the contributions of the experts, constituted a new version, unlike the initial one, as shown in Table 2.

Most of the work came in the third consultation, in which the experts shared the overall effectiveness of the measures associated with each line of action and within each line of action and area. It had the following objectives:

1. To validate the measures designed for each area.
2. To incorporate other measures that the group of experts proposed.
3. To arrive at a final design of the intervention measures, through the positions of the experts towards each measure.

A complete set of measures was proposed organized into the three lines of action and the six areas agreed upon in the earlier phase. Each Area has a number of measures that fluctuated between 8 and 16. In this consultation phase, the experts were asked to state their positions and opinions for each of the proposed measures, through their responses to three questions:

- Would the expert include this proposal as a necessary measure to be applied to the retirement and aging processes of WID?
- What degree of importance would they attach to the measure on a scale from 1 to 5?
- What comments might you add in that regard?

In a final phase of the work to validate the measures, the fourth and final consultation took place, which had the following objectives:

1. To present the final set of intervention measures, which resulted from the contributions of the experts and the work of analysis and categorization of the investigators.
2. To obtain the final approval of the proposal for intervention measures.
3. To prepare a proposal for recommendations that will guide the application of the validated measures towards real work environments.

The experts received the final and previously validated set of measures, to which they could add their final global comments of a general nature. This phase also pursued one of the most significant objectives in relation to the utility of the set of intervention measures: the preparation of a guide with 10 recommendations. In that way, the measures could be applied in work settings, thereby obtaining a practical tool that may be transferred to firms, organizations and public administrations. The experts were expected to share their opinions on each measure, assessing its utility and reasoning their opinions:

## 3. Results

This investigation resulted in a proposal for a compendium of feasible and realistic measures that arose from consensus and the objectivization of expert perspectives, which can be used to assist PID who are aging, guiding the intervention of employers and other workers in the firm; and families and organizations caring for the disabled in Spain.

After the first consultation, all the experts made clear their agreement with the proposed areas that received scores higher than 4 on a scale of 1 to 5. The result of this first consultation was to maintain the areas that had initially been proposed, although slight changes were made to their definitions, such as the consideration of health from a global perspective, which integrates physical, mental and social health, as well as the incorporation of a new concept related with the economic situation of the workers.

Having validated the structure of the areas in which intervention measures were subsequently incorporated following the second consultation, the third consultation was used to validate the measures proposed to the experts, almost all of which were accepted. Any measures that over half of the experts opposed were removed as were those that, despite receiving more *YES* than *NO* scores, obtained scores lower than 3 on the scale of average scores (scale of 1 to 5).

**Figure 1:**
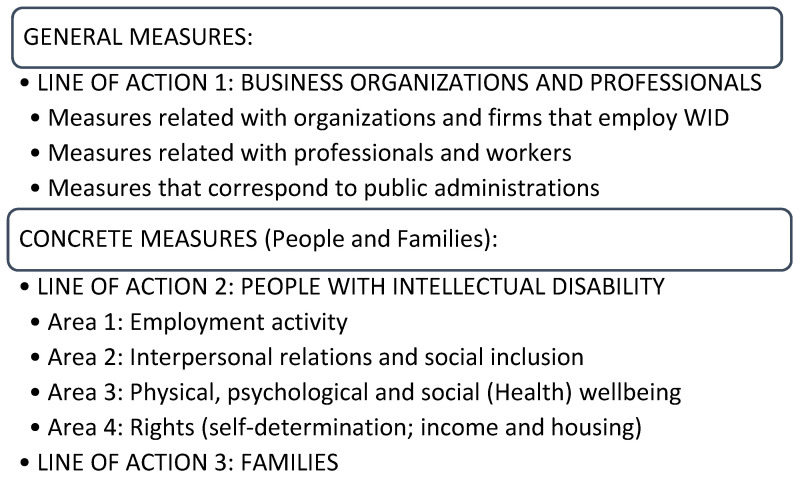
Final schema of the measures.

The final result contained 68 measures, distributed across three lines of action and a set of 10 recommendations for their application. Figure 1.

The first line of action (Professionals and organizations offering support services to people living with disability) contributed the results of the most general measures that firms and organizations that employ WID with support needs should take into account to facilitate their aging process, in an attempt to maintain a high quality-of-life index Table 3 In all, 13 measures were obtained. In this first section, measures were collected that transcended the person with disability and their families. These measures placed the focus on the work and the family environment, important in the definition of their disability within the framework of the social model. Measures organized into three key contexts were presented: measures to implement within firms and disability self-help and social organizations (8 measures); measures that affect professionals (3 measures); and, measures that correspond to the public administrations (2 measures).

The second line of action covered the results of the measures that directly affect WID. In all, 49 measures were proposed, organized into four areas.

The first one concerned the actions to be implemented in the work environment, as listed in Table 4. The levels of consensus and the design obtained in this area, incorporated the final results of all the measures scored between 4.8 and 4.5 points (on a scale of 1 to 5). The measure, Maintain aging workers suffering from (physical and mental) deterioration for as long as possible in employment, postponing retirement from work and extending working life as far as possible up until the age of retirement, was considered inadequate and was therefore removed.

The contributions from the experts were the need to insist on applying initiatives to Know the degree of labor-related satisfaction: motivation, agreement, contributions to work; and Facilitation of measures for relations between fellow workers: breaks, festive occasions and labor-related events.

With regard to the second area: interpersonal relations and social inclusion, the results led to the removal of two measures Table 5: Train specialists in leisure for WID who are aging or suffering from deterioration, who can develop activities that promote active aging: outings, holidays, workshops and physical activities; and Establish entertainment and training programs in communication skills and co-existence with peers that facilitate adaptation to the changes that occur in this new stage of life. In both cases, it was considered that other measures already existed that included similar aspects for preparing PID for retirement.

There were new contributions from the experts within this area. The coincidence may be noted with the aforementioned measure and Setting up a system for the detection of needs, expectations and preferences of older people with respect to the topic of leisure and free time, which was incorporated as a new measure and proposed to the experts for inclusion in the final version. As the development of the measures for the work environment advanced, the inclusion of the following measure was proposed: Maintain the link with fellow workers, especially in the post-employment stage. The following table covers the measures that were agreed within this area.

With regard to the third field: physical, psychological and social wellbeing (Table 6), the experts coincided in maintaining all the proposed measures. The scores given in this field were below the averages of the two earlier ones, especially highlighting the need to generate tools used to measure the deterioration of the workers and to be able to anticipate the implementation of measures.

There were various contributions from the experts aimed at strengthening the development of functional skills that improve the wellbeing of workers with intellectual disability.

Measures to be implemented in the field of legal rights were collected, in order to finalize this second line of action Table 7. Thus, with regard to housing, there was unanimity in the responses concerning the assessment of future systems of coexistence of the PID that, as a consequence, led to the removal of two measures: Promote the establishment of residences specialized in aging and intellectual disability, which may house people having accepted retirement, and to which cases may be referred that, because of greater need for support, require more specific attention. Organize groups of older people with intellectual disabilities and deterioration; and Take steps so that older people once having retired reside in residential homes alongside older people with no disability, receiving adequate treatment and in specialized settings for older people.

A wide variety of opinions were presented in response to the measure: Call for the launch of a new residential model dedicated to PID who are aging and their families, equally affected by aging processes. The debate among the experts led to the decision to maintain it, although incorporating an indication in its final proposal to assess it solely in cases where personal and family circumstances made it advisable.

The third and last line of action of the model, the line of action of Families, covered the results that the experts, through their scores, considered most important in relation with this group that is key in the retirement process of WID. The experts coincided over the need for WID to be involved in the process of change in the employment status of their family, first receiving all the information on the circumstances of their children or siblings, and second taking part in the decision-making. All the measures were maintained, except for Promoting intergenerational activities between older PID, their families and other younger families, promoting their involvement in working activities, but also in other sorts of cultural, social and community initiatives, which the majority of the experts rejected.

In all, 6 measures were proposed for this line of action that can be grouped into two large blocks.

The first covers those measures that have as their objective to guarantee active involvement in the retirement process of families and in the application of changes in the working relations of their family member with disability.

Those measures that, in a second block, were precisely aimed at minimizing the effects that the evident aging of the progenitors has on the person with disability who is working and on that person’s family, through the detection of their needs that will certainly impact on the PID Table 8. Measures are proposed that are aimed at families, so as to alleviate those effects (systems for “breathing spaces” for carers, for example); and the analysis of the impact of aging on families and its consequences.

We conclude the presentation of the results of the investigation with some details of the recommendations needed for the proper application of the set of intervention measures. These recommendations can guide professional practice and can be replicated in the context of the workplace. From our investigation, the need may be inferred for employers to heed the following recommendations on the aging of WID that form their staff:

1. Set up technical teams with training on aging and deterioration of PID in employment and make them available to firms.
2. Conduct evaluations within working environments of the labor-related competencies of workers (360° evaluation) as well as health-care, social, leisure and housing needs.
3. Guarantee the active participation of PID in the process and in decision making.
4. Welcome the involvement of all the interest groups, especially the family.
5. Consider the need to have or to have access to previously prepared and validated working protocols that guarantee the effectiveness and the efficiency of the process and the resources that are employed.
6. The formation or the possibility of attending a committee in charge of following up the process and its evaluation.
7. The incorporation of the topic of aging, its analysis and management of this question in the strategy of the organizations and firms that are employers, with the direct involvement of their managers.
8. The ad hoc adaptation of the measures to each reality, implementing individual plans according to the contextualized situation of each person.
9. The design and the application of measurement indicators, both process and results-related, which yield evidence on the impact that is achieved.
10. The diffusion of good practice within the area of the sector-specific entities, the circles of human resources and in relation to the public administrations.

## 4. Discussion

The numbers of PID have increased by two thirds over the last decade, a situation in which planned support is needed, so as to facilitate the transition to retirement and wellbeing from the start of this new stage in life. In line with [38], we consider that planning the transition from the work stage to retirement, especially among PID, is important, as this collective requires specific support to ensure integral wellbeing beyond merely financial, with a psychosocial focus and promoting an active and socially inclusive style of life [55].

We are in this sense in agreement with [56], concerning the importance of ensuring a balance between participation in group activities and the possibility of maintaining privacy when participating in others, as well as the need to identify the maintenance of interpersonal relations as a quality-of-life indicator that is for some authors more important than financial security.

In harmony with [14,56], our results provide evidence of the need to intervene within a “model of ecological aging”, which implies incorporating interest groups in the design and the planning of interventions for WID who are aging.

Along these same lines, the work in [17] proposed a series of measures to apply with workers who start to suffer premature aging, centering on employment activity, but also on the families and on the organization itself.

Other studies and investigations also share this criterion. [40] looked at that perspective when considering other areas such as leisure and housing, setting out the necessary coordination between bodies, entities and administrations. The opportunities for participating and enjoying leisure activities is the principal predictor of quality of life at the stage of retirement.

In a study completed for FEAPS, organization of entities to support intellectual disability in Spain, [12] proposed up to 52 measures related to healthy aging that likewise contribute a multidimensional approach.

The purpose of these measures coincides with the priority aspects highlighted in [57] to support WID who are aging: to compensate the consequences arising from the aging processes, which are often prematurely initiated, and the transition from employment to retirement. In the same way as [58], these authors were committed to the implementation of support measures, coinciding with our proposal, based on the social model of aging. Rather than solely centered on the job market they were related to the other areas or dimensions involved with quality of life, in line with the social model of disability [49].

The conclusions of the panel of experts in our investigation have shown how the first milestone towards the healthy aging of WID consists of taking the most accurate decision on when they should leave work. On occasions, as [38] affirmed, aging itself obliges WID to leave their employment in the absence of plans to ensure that they can continue working.

The question was proposed as to whether it is acceptable to maintain WID in active employment, despite the emergence of relevant symptoms of aging, moving the end of their working life as far as possible towards the normal age of retirement. This idea was also proposed in the work of [17], who insisted on trying to avoid any situation of double discrimination that might arise following forced retirement. However, and despite the majority of experts insisting upon guarantees for the continuity of living space, care arrangements and relations, the opinions that were collected make it evident that the decision should be taken by the disabled person in the light of an analysis of each situation. The experts warned that, in the first place, their work skills should be evaluated and immediately afterwards the impact of their decision on the closest environment. In this question, the individualization of the processes and respect for life-long learning projects is in line with the contents of [59].

Other measures such as greater flexibility in labor relations (low performance contracts, partial working days, flexible working days) are widely supported among the experts and are in agreement with the majority of authors that were consulted [14,17,60].

However, there are other aspects where any such agreement is not forthcoming. The results of [14] concluded that it was advisable to set up units in work settings to assist the transition from work to retirement, in which to place workers suffering from symptoms of psychological and mental deterioration.

Our results lead us to question this alternative, with which [12,16] also differed. Measures such as the aforementioned one can, in the opinions of the experts who were consulted, have disastrous consequences for the self-esteem of workers, leading to isolation and a negative impact on the working climate.

Moreover, we understand that it would be necessary to approach a new line of study centered on promoting measures in relation to employers and working conditions. These include the need for labor assessment, claims for improvements and changes to employment positions, working relations, trade union affiliations and access to legal advice, which although not having arisen in the expert consultancy process, are very important aspects to take into consideration.

The aging of workers with disability also affects their families, as was also indicated in [61], as the family was a key factor in the promotion and the development of healthy aging, in line with [62]. We share the opinion of [63], in so far as the families face an aging process and that, added to the physical deterioration of the parents, there is also parental uncertainty towards the future of their children and the impact that their absence will have among those who take up the role of carers, which in most cases corresponds to the siblings.

At present, we can affirm that there are neither specific resources nor measures to assist with the aging of WID and even more so no other measures provide them with quality services once they have retired from employment, a point raised by many other authors [15,19,64].

Despite the interest aroused over recent years and the incipient yet growing demand of the self-help social movements of disabled people to find an answer to this problem, the topic has surprised an unprepared political class, as other authors have noted [45,46]. Having found no post-employment alternatives in response to the new needs of ex-WID, the design of valid alternatives and their proposal to public administrations is converted into an urgent line of work. Many of the experts who were consulted have highlighted this aspect, especially those linked to the area of management of firms and organizations that employ them. Along these lines [54,55], they affirmed that the impact of aging on the quality of life of WID calls for action on resources, equipment and the degree of specialization of the professionals who are involved, without overlooking the families, who constitute the true source of assistance. Demands that may be added to the demands of the main carers in the form of pensions, remuneration or services and specific support, as [65,66] have also mentioned. Moreover, one line of work reported in this study is centered on setting up normative guidelines that favor the introduction of such measures at a general level, a need that other authors [67] have also pointed out and with which we coincide.

## 5. Conclusions

Faced with the need to propose alternatives that guarantee active aging and the quality of life of WID, the proposed study has responded to the objective of proposing a model for attention to the active aging of WID, which is also transferable to public policies.

The results have highlighted the need to propose alternatives and policies that guarantee active ageing; the importance of intervening within the work setting following the appearance of the first symptoms and the necessary participation in the process of the workers who are affected and their families.

In all, 68 measures have been obtained, grouped into three lines of action with an impact on the lives of aging workers: in the field of firms and organizations that are employers; among the PID and in the family setting. Measures were grouped into essential areas such as health, housing, leisure and interpersonal relations. Ten recommendations have been drawn up, focused on firms and organizations, in order to implement the proposed measures.

Aging and premature deterioration of WID are an immense challenge today, which should form part of the strategy of organizations and public administrations with responsibility for these matters. It is considered essential to approach this question, which brings with it significant social and political consequences and may in the future be turned into a problem with a difficult solution.

The principal contributions in the area of business organizations that employ PID and their professionals refers to the need to generate protocols for the retirement of workers and to approach questions in a coordinated manner in all relevant areas; as well as the need for organizations to have diagnostic and evaluation systems for the aging indices of their workers; and to guarantee the training that professionals need to approach this phase throughout the lives of PID.

It is also proposed to emphasize the need to lend special attention to caring for health, insisting on the need to contemplate it in its threefold dimension of physical, mental and social wellbeing.

Finally, the contribution of the experts may be highlighted, in that they reinforced the idea of continuity of attention and relations that the workers have maintained over previous years, and setting up mechanisms that guarantee both access to housing and economic independence, once active working life ends.

Two key moments in the intervention may be noted. The first, during the last phase of working life, when the WID remain active, guaranteeing their quality of life. In the second place, planning the retirement process. This question requires the active interest of both the disability sector and the public administrations, as well as the investigation of possible alternatives that reveals itself as a key and necessary field of intervention.

In the case of the premature aging of workers with intellectual disability, an intervention is considered essential from the time at which the first evidence of deterioration is produced. Emphasis is placed on the obligation of entities and firms to generate systems to detect cognitive, emotional and physical deterioration that can provoke problems related with employment activity and worker safety.

The measures that are necessary to approach this question should set out an integral and multidisciplinary approach. They should underline a fundamental aspect that is the individualization of the measures, adapting the previously established protocols to the peculiarity of each worker. The active participation of the individual in the process is especially important; deciding when to retire, accepting the measures and choosing the conditions for a life project following retirement from work.

In this sense, one line of action might consist in encouraging a change of attitude among social care organizations and professionals with respect to the employment opportunities for PID and their aging in general.

It is concluded that an essential principal that can guarantee quality of life is the “continuity of care services” and no disconnection with the reference settings. In this sense, the maintenance of the job must be guaranteed when detecting symptoms of deterioration, with the incorporation of as many measures for the adaptation of posts and flexible working relations, as may be necessary: reductions of working life, changes in job posts, a slower pace of work, and adaptation to the tasks.

We consider that the majority of the resulting measures may be applicable and valuable for all workers and not only for the group with disabilities.

Finally, it was concluded that it is essential to rethink and to recast a post-employment model, financed by the public administration from a portfolio of services for supporting dependency, adjusted to the new peculiarities of PID who have followed an integrated working career in their lifetime, who are economically independent and have high levels of independence. The lack of resources may be generating an unnecessary extension of the working relation and, in many cases, bringing the return of a resource now abandoned for this group of people: occupational or day care centers which, in our opinion, are not the most suitable place for active aging, because they have an aspect of occupational activity that is related or focused on people with important dependencies and physical and cognitive deterioration. It is not always a true reflection of the necessities and the life cycle of PID who can undertake more complex occupational activities. On this point, a debate is opened on the role that grants and benefits from the government and the regional administrations can play to perpetuate this type of sheltered occupational work, which is necessary to stimulate their employment. A resource should be devised for a new social category: PID of a mature age, in active retirement. This aim is, in our understanding, a line of work for subsequent years.

The results of our investigation have led us to explain some of its limitations and some lines of future research. The proposed measures have yet to be implemented in concrete ways, as well as evaluation indicators of the results with which to measure their impact on workers, firms, families and professionals.

Moreover, we believe that the measures proposed in this paper are applicable to the reality of many firms. Although they have yet to be implemented in real contexts, they constitute a phase that is at present ongoing: the implementation of the model, the assignment of applicable indicators and the evaluation of the effectiveness of the measures that have been presented in this paper.

## Figures and Tables

**Table 1:** Proposed areas.

Aera	Brief Explanation of the Area	1–5
1. Measures related with employment activity.	Refers to everything that we should do, in order to improve the quality of life of Workers with Intellectual Disability (WID) during the last phase of their active employment, without the effects of aging interfering negatively in their lives; on their productivity; and within the general working climate.	
2. Health-related measures.	Refers to everything that should be taken into account during the aging process of the workers. Preventive measures and specific interventions.	
3. Housing-related measures.	Refers to how to approach the question of cohabitation, when and with whom to live and how to contemplate this field after working life.	
4. Measures related with Leisure, enjoyment of free time and interpersonal relations.	Refers to whether they are or are not necessary and, in any case, which specific measures or activities for workers with intellectual disability are necessary when they reach old age, paying attention to the important dimension of interpersonal relations for their quality of life.	
5. Measures related with the exercise of the Rights of people with disability.	Refers to how to respect the rights of WID in the fundamental areas of self-determination, decision-making capability and all other rights as citizens.	
6. Measures and actions directed towards and with the Families of WID.	Refers to how to support families in this process and how to work with them to help their children or siblings with the changes that leaving employment might entail.	
7. Measures related with Organizations and firms that contract Professionals.	Refers to the aspects and strategies that should be worked upon within organizations, the firms that employ PID and their workers and professionals, in order to approach the new necessities of PID and aging before, during and after the employment relations end.	
8. Actions and interventions to take into account during the Process of Leaving Employment.	Refers to how this transitional process to non-activity should be planned, in order to guarantee the quality of life for WID.	

**Table 2:** Schema of lines of action and areas.

**LINE OF ACTION 1** People Living with Disabilities	1. Work area	
	2. Area of social relations and social inclusion	
	3. Area of physical, psychological and social wellbeing	
	4. Area of rights	4.1. Self-determinationand capability to choose
		4.2. Housing
		4.3. Minimum income and acceptable of living standars
**LINE OF ACTION 2** Families	5. Measures that help them and their closest family (children, siblings, and others) participate in the process of ending working life and leaving employement.	
	6. Measures fod support to the families.	
**LINE OF ACTION 3** Employers (Organizations) and Professionals	7. Interventions in the dynamics of organizations and firms.	
	8. Measures with and for professionals providing support services	

**Table 3:** Results for line of action 1.

Measures That Correspond to Organizations and Firms that Employ People with Intellectual Disability (PID) and Their Work Systems
1. Set up a working group to approach the question of aging of their workers.
2 Design an Aging and Worker Deterioration Intervention Protocol in firms with procedures, measures, agents and impact indicators both for the person and the organization.
3. On an annual basis, conduct population studies and aging tendencies of their staff.
4. Apply diagnostic systems of the degree of aging and deterioration of the staff.
5. Prepare a personal aging support plan that should include the plan for intervention in employment (measure 2.1.3.) and other transversal measures (housing, leisure, social and family relations).
6. Set up a legal assessment service: economics and legal rights.
7. In the Area of organizational follow up, integrate training actions and preparation for a new stage of life once the working relation ends, targeting workers with intellectual disability (WID).
8. Lead and participate in the organization of services jointly funded through the public administrations.
**Measures that Affect Professionals Supporting WID**
9. Improve the skills of professionals supporting WID within firms.
10. Draft manuals of good practices, materials and guides of minimum standards.
11. Pay special attention to personal and labor-related wellbeing of professionals, evaluating the degree of satisfaction and the need for both technical and human support.
**Measures that Correspond to the Administrations**
12. Establish channels of coordination and communication between organizations, administrations and bodies committed to working with WID.
13. Incorporate joint services through which post-employment support services may be managed in the portfolio of services assisting WID.

**Table 4:** Measures from line of action 2, referring to the work environment.

Measures Oriented Towards Improving Work-Related Activity
1. Avoid situations in which workers with deterioration remain active in their jobs and feel obliged to remain until retirement, if this situation is negatively impacting on their quality of life.
2. Implement an evaluation system of their working competences. Evaluate and follow up the deterioration that may occur over time on an annual basis.
3. Draft an employment intervention plan that includes all necessary changes that might have to be applied in employment-related matters and working conditions.
4. Maintain workers who are aging and maintain themselves active in the employment-related conditions that have become habitual for them, with their fellow workers, without introducing ad hoc groupings based on age and deterioration.
5. Ensure that labor relations are flexible, applying in each specific case, as detailed in the Employment Intervention Plan (measure 3): working day reductions; part-time contracts; rest times when the labor activities are interrupted; modifications of working hours; slower pace of work; additional therapeutic breaks.
7. Analyze the degree of satisfaction of the worker with disability with the remunerated employment activity, on a permanent basis and at least once a year.
8. Implement training workshops and training in healthy habits at work that minimize the possible effects of having acquired or succumbed to bad practice for reasons of age or deterioration.
9. Guarantee the principle of universal accessibility within the workplace and design for everybody, ensuring that work spaces, instruments, and work tools, instructions, etc. are acceptable for the capabilities of WID and ensure inclusive environments for aging people while at work.
10. Intensify evaluation plans and the prevention of occupational risks and increase training systems for risk prevention adapted to WID aging at work.

**Table 5:** Line of action 2, referring to social inclusion.

Measures Oriented towards Improving Interpersonal Relations and Social Inclusion
1. Apply instruments to detect needs, expectations and preferences for leisure time, with a view to planning and the provision of acceptable levels of support as WID may require.
2. Offer new leisure activities within a new model that is developed within community settings, together with the rest of the population, following the paradigm of inclusive leisure.
3. Include an offer of activities targeting aging WID in local and community plans for active aging.
4. Propose specific measures (on leisure, sport and holidays) within organizations, following the principles of inclusion, as mentioned in Article 30 of the UN Convention on the rights of persons with disabilities.
5. Design and set in motion all measures that promote constant links with the family environments of PID, especially in view of the demise of parents and the assumption of guardianship and care of the second generation.
6. Facilitate measures that can maintain links with fellow workers, especially in the post-employment stage and throughout the transitional process of retirement.
7. Promote peer-based relations through alternatives such as self-help groups among older people that have passed through the retirement process.
8. Develop activities that promote the inter-generational relations of aging WID and other people with disabilities of a younger age.
9. Generate a new category of “retired volunteers”: older people with no disability who interact with aging workers with intellectual disability, helping them to adapt to group situations with a certain generational similarity.

**Table 6:** Measures for line of action 2, referring to health care.

Measures Oriented towards Improving Health
1. Design an instrument with which to detect physical, cognitive and behavioral symptoms associated with aging and deterioration among WID.
2. Incorporate medical and psychological reviews, through the application of instruments adapted to measuring the wellbeing of WID suffering from deterioration regardless of their age.
3. Monitor the mental health of WID who are aging and WID suffering from deterioration.
4. Pay special attention to cognitive deterioration and to the prevention of dementia in the aging process and the deterioration of WID.
5. Apply health campaigns designed for the population in general to PID who are aging.
6. Generate, in coordination with the Administration, a framework health-care model for WID who are aging.
7. Improve the training of health professionals, for the incorporation of key aspects on the peculiarities that characterize PID and their aging process.
8. Provide training on the aging process of PID to interest groups in their immediate environment: their families, health-care professionals and people planning their activities at health centers.
9. Establish training programs on health care habits, targeting WID, strengthening the development of functional skills that improve their wellbeing.
10. Prepare WID for old age through training in emotional skills that facilitate responses in the face of stressful situations that might arise at that age: death, mourning, changes in their life or leaving employment.
11. Demand coordination of socio-health care as an essential part of health care for WID.

**Table 7:** Measures for Line of Action 2, referring to the guarantee of rights.

Measures Oriented towards Guaranteeing Rights
1. Guarantee the participation of PID in the process of leaving employment, incorporating it from the outset, and actively participating in decision making.
2. Design the necessary support systems that guarantee the participation of PID and the expression of the preferences throughout the process.
3. Guarantee quality, general and community services and specific services after leaving employment, under the modality of “continuity of care”.
4. Encourage the participation of PID in the choice of services, programs and activities in which to participate, thereby guaranteeing the right to self-determination and decision-making.
5. Update and periodically review, as from 45 years of age, the assessment of the disability, with the objective of modifying the assigned percentages, as a function of the change in deterioration due to age.
6. Inform PID of their rights, assuring them that they are fully understood.
7. Guarantee the right to privacy in general and to intimacy and to development of the sexual identity of WID, providing them with support for a full and a healthy sexual life.
8. Ensure flexible conditions for early retirement.
9. Regulate the possibility of accessing state aid and compensating reduced income when reductions in working life take place, through access to benefits to ensure a minimum income.
10. Guarantee the right of PID who decide to retire to decent conditions of life, through the establishment of guaranteed income payments.
11. Design and apply validated tools that, on the one hand, detect the skills and, on the other, the support needs, in order to guarantee a decent life with no risks for health or personal quality of life.
12. Ensure that PID can choose to stay in their habitual social setting, during the last stage of their occupational life when leaving employment, incorporating adaptations of space, hours and times.
13. Arrange housing/supervised apartments (not residences), in which PID can as they age and leave employment coexist with peers, respectful of their choices to share their life with particular people, avoiding in so far as possible excessively institutionalized settings.
14. Promote the figure of the “personal assistant” who facilitates support with the activities of daily life, which permit integrated, peaceful co-existence with zero conflicts, and that contribute to the quality of life of PID.
15. Respond to the new needs of people who live alone and for whom the process of deterioration can affect their independence.
16. Facilitate the possibility of experiences of coexistence among older PID together with their parents, equally affected by aging processes. This option is only to be valued in cases where personal and family circumstances make it advisable.

**Table 8:** Measures for line of action 3: the family.

Measures Aimed at the Family Environment
1. Involve families in the process of adaptations and retirement from employment of WID.
2. Set up information programs aimed at families on the shortcomings associated with the aging of their children/siblings, mitigating the effects of aging that interfere with family dynamics.
3. Design and apply awareness raising sessions on the future retirement of their family member with disability in the framework of the model for assistance to families.
4. Detect the needs of families through ad hoc scales, faced with a situation of double aging, through coordinated interventions targeting children and/or siblings who are aging.
5. Work on the design of innovative services targeting families (parents and siblings) of the PID, all of whom are undergoing aging processes: establish systems for ‘breathing spaces’, targeted support, and training programs, among others.
3.6. Study the impact of aging PID on their families.

## Data Availability

Data sharing not applicable. No new data were created or analyzed in this study.
